# Downregulation of miR-10b-3p by EBV promotes tumor growth and metastasis via *ITGAV* in nasopharyngeal carcinoma

**DOI:** 10.1371/journal.ppat.1014304

**Published:** 2026-06-09

**Authors:** Yu Zhang, Yaoqiang Shi, Yingdong Zou, Li Li, Ziqin Dian, Yuling Chen, Hang Zhao, Jiajun Wang, Yi Sun

**Affiliations:** 1 Department of Clinical Laboratory, The First People’s Hospital of Yunnan Province, Kunming, Yunnan, China; 2 Institute of Basic and Clinical Medicine, The First People’s Hospital of Yunnan Province, The Affiliated Hospital of Kunming University of Science and Technology, Kunming, Yunnan, China; 3 Department of Clinical Laboratory, The Yunnan Provincial Hospital of Traditional Chinese Medicine, Kunming, Yunnan, China; 4 Yunnan Medical Center for Pediatric Diseases, Yunnan Institute of Pediatrics, Kunming Children’s Hospital, Kunming, Yunnan, China; 5 Kunming Key Laboratory of Children Infection and Immunity, Yunnan Key Laboratory of Children’s Major Disease Research, Yunnan Province Clinical Research Center for Children’s Health and Disease, Kunming, Yunnan, China; 6 Medical School, Kunming University of Science and Technology, Kunming, Yunnan, China; University of Utah, UNITED STATES OF AMERICA

## Abstract

Nasopharyngeal carcinoma (NPC) is a malignant epithelial tumor strongly associated with Epstein-Barr virus (EBV) infection. EBV-mediated dysregulation of host microRNAs (miRNAs) contributes to NPC pathogenesis, but the functions of many EBV-regulated host miRNAs remain incompletely defined. miR-10b-3p is markedly downregulated in EBV-positive NPC, yet its biological significance and downstream mechanism remain unclear. Here, we found that miR-10b-3p was reduced in EBV-positive NPC tissues and was further suppressed following EBV infection of non-malignant nasopharyngeal epithelial cells and EBV-negative NPC cell lines. Restoration of miR-10b-3p expression markedly inhibited cell proliferation, colony formation, migration, invasion, and epithelial-mesenchymal transition (EMT) in EBV-positive NPC cells, whereas inhibition of miR-10b-3p in EBV-negative NPC cells produced the opposite effects. In nude mouse xenograft and lung metastasis models, overexpression of miR-10b-3p significantly reduced tumor growth and pulmonary metastasis. Mechanistically, miR-10b-3p directly targeted the 3′-UTR of integrin subunit alpha V (*ITGAV*), leading to decreased ITGAV expression and subsequent attenuation of STAT5 and ERK1/2 signaling. Forced ITGAV expression partially reversed the suppressive effects of miR-10b-3p on tumor cell proliferation, migration, invasion, and EMT. Moreover, miR-10b-3p levels were inversely correlated with ITGAV expression in NPC tissues. Collectively, these findings identify an EBV-regulated miR-10b-3p/ITGAV/STAT5-ERK1/2 axis in NPC and show that loss of miR-10b-3p promotes tumor growth and metastasis by relieving *ITGAV* repression, suggesting potential therapeutic targets for EBV-associated NPC.

## Author summary

Nasopharyngeal carcinoma (NPC) is a virus-associated cancer closely linked to Epstein-Barr virus (EBV) infection and remains a major health burden in endemic regions. Although EBV is known to alter gene regulation in infected cells, the roles of many EBV-regulated host microRNAs remain unclear. This study shows that EBV suppresses miR-10b-3p, a small regulatory RNA that helps restrain tumor cell growth and spread. Loss of miR-10b-3p enhanced NPC cell growth, migration, invasion, and metastasis-associated features, whereas restoration of miR-10b-3p reduced tumor growth and lung metastasis in experimental models. Integrin subunit alpha V (*ITGAV*) was identified as a direct target of miR-10b-3p. Suppression of miR-10b-3p increased ITGAV expression and activated downstream signaling pathways that promote tumor progression. These findings reveal a previously unrecognized EBV/miR-10b-3p/ITGAV axis in NPC and suggest that restoration of miR-10b-3p activity or inhibition of ITGAV-dependent signaling may provide potential therapeutic strategies for EBV-associated NPC.

## Introduction

Nasopharyngeal carcinoma (NPC) is a malignant epithelial tumor arising from the mucosal lining of the nasopharynx, typically originating in the pharyngeal recess [1,2]. According to global cancer statistics, approximately 129 000 new cases of NPC were reported in 2018, accounting for about 0.7% of all newly diagnosed cancers [1]. When detected at an early stage, NPC responds well to radiotherapy, achieving an overall five-year survival rate that exceeds 80% [3]. However, nearly 70% of patients are diagnosed at advanced stages [4,5], resulting in poorer clinical outcomes and substantial treatment-related morbidity. Consequently, elucidating the molecular mechanisms underlying NPC initiation and progression remains essential for improving early detection and therapeutic outcomes.

Epstein-Barr virus (EBV), a ubiquitous member of the *Herpesviridae* family, plays a central role in the pathogenesis of NPC [6,7]. More than 95% of NPC patients exhibit serological or genomic evidence of prior EBV infection [8,9]. EBV contributes to oncogenesis in part through the dysregulation of host and viral microRNAs (miRNAs), which are small non-coding RNAs that regulate gene expression at the post-transcriptional level [10]. During latent infection, EBV expresses a cluster of its own miRNAs, known as BamHI-A rightward transcripts (BARTs), while concurrently altering the expression of host miRNAs [11,12]. This EBV-mediated reprogramming of miRNA networks profoundly influences signaling pathways involved in cell proliferation, apoptosis, and metastasis, thereby facilitating malignant transformation [13–16]. Although bioinformatic and functional studies have indicated that EBV-induced miRNA dysregulation contributes to NPC development [16,17], the biological functions and downstream mechanisms of many EBV-regulated host miRNAs remain insufficiently characterized.

The miR-10b locus generates two mature strands, miR-10b-5p and miR-10b-3p, which have distinct seed sequences and may regulate different target repertoires. Previous studies in EBV-associated NPC have mainly focused on miR-10b-5p, which can be induced by the EBV oncoprotein latent membrane protein 1 (LMP1) and has been implicated in metastasis through a Twist1-associated program [18]. By contrast, miR-10b-3p has been reported to be consistently downregulated in EBV-positive NPC biopsies [19]. However, its biological function, direct downstream targets, and role in EBV-associated NPC progression remain largely undefined. Therefore, miR-10b-3p warrants independent investigation as an under-characterized strand of the miR-10b locus in EBV-associated NPC.

The present study was designed to define the biological role and downstream mechanism of miR-10b-3p in EBV-associated NPC. Using 30 EBV-positive NPC specimens and 30 demographically comparable non-malignant nasopharyngeal specimens, together with gain- and loss-of-function models in endogenously EBV-positive C666-1 cells and experimentally EBV-infected EBV-negative NPC cell lines, this study examined the EBV-associated suppression and functional consequences of miR-10b-3p dysregulation. Restoration of miR-10b-3p reduced NPC cell proliferation, migration, invasion, and epithelial-mesenchymal transition (EMT) *in vitro*, and attenuated tumor growth and lung metastasis *in vivo*. Mechanistically, integrin subunit alpha V (ITGAV) was identified as a direct 3′-UTR target of miR-10b-3p, and *ITGAV* repression was linked to reduced STAT5 and ERK1/2 signaling. Rescue experiments using forced ITGAV re-expression further supported ITGAV as a key downstream effector of miR-10b-3p-mediated tumor suppression. Collectively, these findings define an EBV-associated miR-10b-3p/ITGAV/STAT5-ERK1/2 regulatory axis in NPC, provide functional evidence for the previously under-characterized 3p arm of the miR-10b locus, and suggest that this axis may provide a mechanistic basis for therapeutic exploration in EBV-associated NPC.

## Results

### miR-10b-3p is downregulated in EBV-positive NPC and suppresses NPC cell proliferation

**Fig 1:**
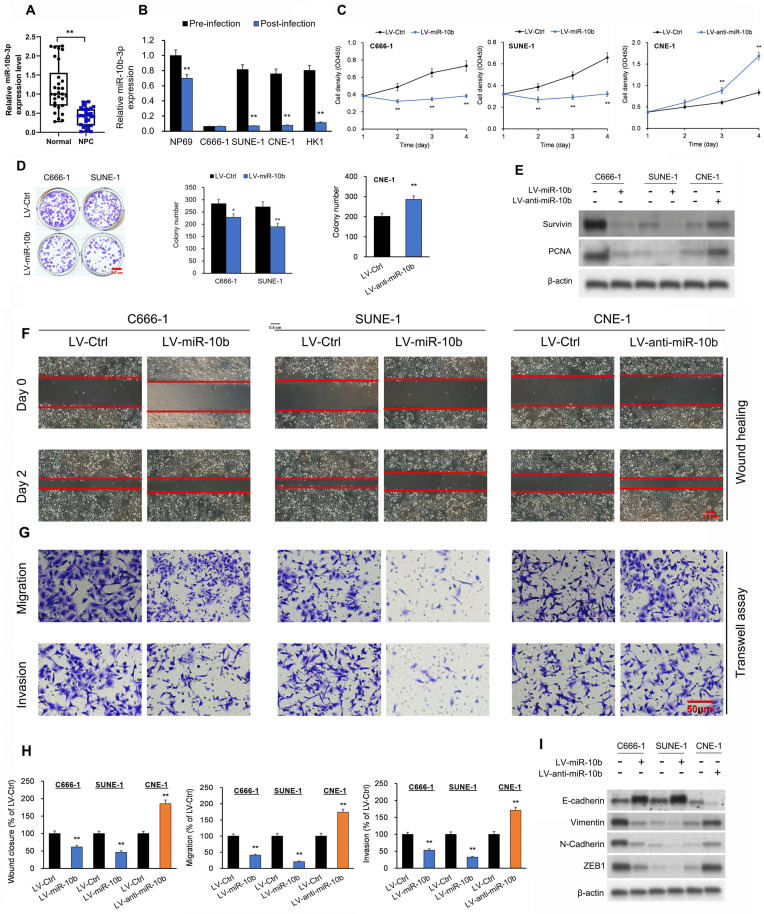
miR-10b-3p is downregulated in EBV-positive NPC tumors and suppresses NPC cell proliferation, migration, invasion, and EMT-associated changes *in vitro.* **(A)** Relative miR-10b-3p expression in EBV-positive NPC tumor specimens (n = 30) and demographically comparable non-malignant nasopharyngeal specimens (n = 30). Data are represented as median ± IQR (boxes) and absolute ranges (whiskers). *P < 0.05, **P < 0.01 vs. Normal [unpaired Student’s t-test]. **(B)** Relative miR-10b-3p expression in NP69 and EBV-negative NPC cell lines SUNE-1, CNE-1, and HK1 before and after infection with the EBV M81 strain. C666-1 was included as an endogenous EBV-positive NPC reference cell line. U6 snRNA was used as the internal control. **(C–I)** Functional assays were performed in EBV-positive C666-1 cells and EBV-infected SUNE-1 cells transfected with LV-Ctrl or LV-miR-10b, and in EBV-negative CNE-1 cells transfected with LV-Ctrl or LV-anti-miR-10b. LV-miR-10b denotes the miR-10b-3p overexpression construct, and LV-anti-miR-10b denotes the miR-10b-3p inhibition construct. **(C)** Cell proliferation was determined using the CCK-8 assay. **(D)** Representative crystal violet-stained colonies and quantification of colony formation. **(E)** Representative immunoblots showing the protein levels of the proliferation markers Survivin and PCNA. **(F)** Representative images of wound healing assays at day 0 and day 2. **(G)** Representative images of Transwell migration and Matrigel invasion assays. **(H)** Quantification of wound closure, migration, and invasion. **(I)** Representative immunoblots showing the expression of EMT-associated markers E-cadherin, vimentin, N-cadherin, and ZEB1. For **B, C, D**, and **H**, data are shown as mean ± SEM from three independent biological experiments, each performed with three technical replicates. In B, *P < 0.05 and **P < 0.01 vs. pre-infection. In **C, D**, and **H**, *P < 0.05 and **P < 0.01 vs. LV-Ctrl.

qPCR analysis of 30 EBV-positive NPC tissues and 30 demographically comparable non-malignant nasopharyngeal specimens revealed significant downregulation of miR-10b-3p in NPC tumors (Fig 1A). These findings were consistent across independent specimens, indicating that reduced miR-10b-3p expression is a common molecular feature of EBV-associated NPC. Similarly, EBV infection of the immortalized nasopharyngeal epithelial cell line NP69 and the EBV-negative NPC cell lines SUNE-1, CNE-1, and HK1 using the EBV M81 strain resulted in a pronounced decline in miR-10b-3p levels, supporting EBV-mediated suppression of miR-10b-3p expression *in vitro*. In contrast, the inherently EBV-positive C666-1 cells [20] showed no further reduction in miR-10b-3p abundance under the same EBV exposure conditions, consistent with a persistently low miR-10b-3p state in cells with endogenous EBV latency (Fig 1B).

To elucidate the functional consequences of miR-10b-3p dysregulation, gain- and loss-of-function experiments were performed. miR-10b-3p overexpression in EBV-positive C666-1 and EBV-infected SUNE-1 cells, and miR-10b-3p inhibition in EBV-negative CNE-1 and HK1 cells, were confirmed by qPCR (S1 Fig). Restoration of miR-10b-3p expression markedly suppressed C666-1 and SUNE-1 cell proliferation and colony-forming capacity, accompanied by significant decreases in the expression of the proliferation-associated proteins Survivin and PCNA (Fig 1C–1E). Conversely, inhibition of endogenous miR-10b-3p in CNE-1 cells significantly enhanced proliferative activity, colony density, and expression of these markers, indicating a direct role for miR-10b-3p in controlling NPC cell growth. Collectively, these results demonstrate that miR-10b-3p is reduced in EBV-positive NPC tissues and is further suppressed following EBV infection *in vitro*. The reduced miR-10b-3p expression correlates with enhanced proliferative capacity of NPC cells, suggesting that miR-10b-3p functions as a tumor-suppressive miRNA in the context of EBV-positive NPC.

### miR-10b-3p inhibits migration, invasion, and EMT in EBV-positive NPC cells

To further determine the role of miR-10b-3p in NPC progression, cell motility and invasiveness were evaluated using wound healing and Transwell assays [21]. Overexpression of miR-10b-3p markedly impaired the migratory ability of EBV-positive C666-1 and EBV-infected SUNE-1 cells, as indicated by delayed wound closure and reduced migration through uncoated Transwell membranes (Fig 1F–1H). Invasion assays using Matrigel-coated chambers further showed that miR-10b-3p overexpression significantly decreased the number of invading cells compared with control groups, supporting an inhibitory effect on invasive capacity. In contrast, inhibition of endogenous miR-10b-3p in CNE-1 cells promoted both migration and invasion, as reflected by accelerated wound healing and increased cell penetration through Matrigel. Quantitative analysis of replicate experiments showed that miR-10b-3p restoration led to a significant reduction in both migration and invasion indices (Fig 1H), supporting its suppressive role in NPC cell motility.

Western blot analysis further showed that miR-10b-3p overexpression increased the epithelial marker E-cadherin and decreased the mesenchymal markers N-cadherin, ZEB1, and Vimentin (Fig 1I). Conversely, inhibition of endogenous miR-10b-3p decreased E-cadherin and increased mesenchymal marker expression, indicating that miR-10b-3p negatively regulates EMT-associated changes in NPC cells. Together, these results indicate that miR-10b-3p suppresses NPC cell migration, invasion, and EMT-associated molecular changes, supporting its inhibitory role in the metastatic phenotype of EBV-associated NPC.

### miR-10b-3p suppresses NPC tumor growth and lung metastasis *in vivo*

**Fig 2:**
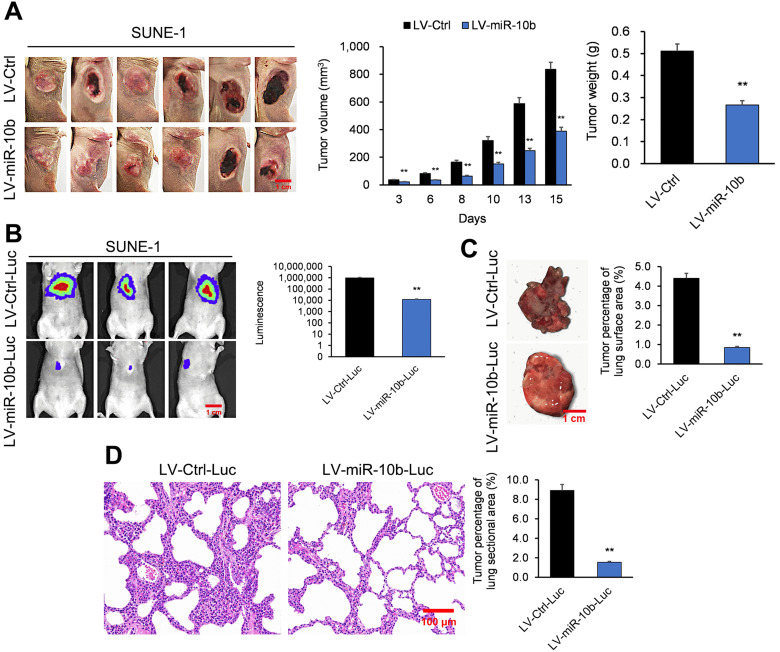
miR-10b-3p suppresses NPC tumor growth and metastasis *in vivo.* **(A)** Subcutaneous xenograft tumor growth in nude mice injected with EBV-infected SUNE-1 cells transfected with LV-Ctrl or LV-miR-10b. LV-miR-10b denotes the miR-10b-3p overexpression construct. Representative gross images of xenograft tumors in mice, tumor growth curves, and final tumor weights are shown (n = 9 mice per group). **(B)** Lung metastasis model established by tail vein injection of luciferase-labeled EBV-infected SUNE-1 cells transfected with LV-Ctrl-Luc or LV-miR-10b-Luc. Representative bioluminescence images and quantification of luminescent signals at week 5 post-injection are shown (n = 9 mice per group). **(C)** Representative gross lung images and quantification of the tumor-occupied percentage of the lung surface area. **(D)** Representative H&E-stained lung sections and quantification of the tumor-occupied percentage of the lung sectional area. Data are shown as mean ± SEM. For the tumor growth curve in **A**, comparisons at individual time points were performed using unpaired Student’s t-test. Final tumor weights in A and quantitative data in **B-D** were analyzed using unpaired Student’s t-test. *P < 0.05 and **P < 0.01 vs. LV-Ctrl or LV-Ctrl-Luc, as appropriate.

To assess whether miR-10b-3p also suppresses NPC tumor growth *in vivo*, a subcutaneous xenograft model was established using EBV-infected SUNE-1 cells with or without miR-10b-3p overexpression. Equal numbers of cells (1 × 10^6^) were injected subcutaneously into the dorsal flank of each female BALB/c nude mouse. Tumors derived from miR-10b-3p-overexpressing cells showed slower growth kinetics and smaller volumes than control tumors (Fig 2A). At the experimental endpoint, the mean tumor weight in the miR-10b-3p group was approximately 50% lower than that of the control group (Fig 2A). To further assess lung metastatic burden, a tail-vein injection model was established using luciferase-labeled EBV-infected SUNE-1 cells expressing miR-10b-3p or the control vector (S2 Fig). Bioluminescence imaging revealed significantly lower signal intensity in the lungs of mice injected with miR-10b-3p-overexpressing cells compared with controls (Fig 2B), indicating a substantial reduction in metastatic burden. Macroscopic observation showed a reduced lung metastatic area, and hematoxylin-eosin staining showed fewer microscopic metastatic foci (Fig 2C–2D). Collectively, these *in vivo* results corroborate the *in vitro* findings and support a tumor-suppressive and anti-metastatic role for miR-10b-3p in EBV-positive NPC.

### miR-10b-3p expression inversely correlates with ITGAV expression in EBV-positive NPC

**Fig 3:**
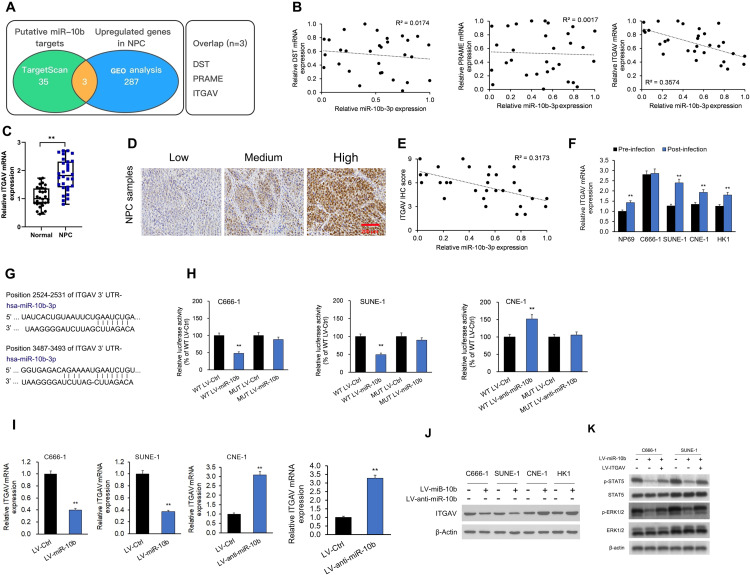
miR-10b-3p directly targets *ITGAV* and suppresses ITGAV-associated STAT5 and ERK1/2 signaling. **(A)** Candidate miR-10b-3p target genes were screened by integrating TargetScan-predicted miR-10b-3p targets with genes upregulated in NPC from GEO analysis. Venn analysis identified three overlapping candidate targets: *DST*, *PRAME*, and *ITGAV*. **(B)** Correlation analysis between miR-10b-3p expression and *DST*, *PRAME*, or *ITGAV* mRNA expression in EBV-positive NPC tumors (n = 30). miR-10b-3p expression was inversely correlated with ITGAV mRNA expression, but not with *DST* or *PRAME* mRNA expression. **(C)** Relative *ITGAV* mRNA expression in EBV-positive NPC tumor specimens (n = 30) and demographically comparable non-malignant nasopharyngeal specimens (n = 30). GAPDH was used as the internal control. Box plots show the median, interquartile range, and minimum–maximum values. **(D)** Representative IHC images showing low, medium, and high ITGAV protein expression in EBV-positive NPC tumors. IHC scores were categorized as low (0–3), medium [4–6], and high [7–9]. **(E)** Correlation between ITGAV IHC scores and miR-10b-3p expression in EBV-positive NPC tumors (n = 30). **(F)** Relative *ITGAV* mRNA expression in NP69 and EBV-negative NPC cell lines SUNE-1, CNE-1, and HK1 before and after infection with EBV M81. C666-1 was included as an endogenous EBV-positive NPC reference cell line. GAPDH was used as the internal control. **(G)** Predicted binding sites of miR-10b-3p in the wild-type *ITGAV* 3′-UTR. Two putative miR-10b-3p binding sites were identified. **(H)** Luciferase activity of WT or MUT *ITGAV* 3′-UTR reporters in C666-1 and SUNE-1 cells transfected with LV-Ctrl or LV-miR-10b, and in CNE-1 cells transfected with LV-Ctrl or LV-anti-miR-10b. **(I)** Relative *ITGAV* mRNA expression after lentiviral transduction with LV-Ctrl, LV-miR-10b, or LV-anti-miR-10b, as indicated, in C666-1, SUNE-1, CNE-1, and HK1 cells. **(J)** Representative immunoblots showing ITGAV protein expression after lentiviral transduction with LV-Ctrl, LV-miR-10b, or LV-anti-miR-10b, as indicated. **(K)** Representative immunoblots showing STAT5 and ERK1/2 signaling in EBV-positive C666-1 cells and EBV-infected SUNE-1 cells transfected with LV-Ctrl, LV-miR-10b, or LV-miR-10b + LV-ITGAV. LV-miR-10b + LV-ITGAV denotes a single dual-expression lentiviral construct expressing both miR-10b-3p and ITGAV. β-actin was used as the loading control. For **F**, **H**, and **I**, data are shown as mean ± SEM from three independent biological experiments, each with three technical replicates. Representative immunoblots in **J** and **K** are from three independent experiments. For **C**, **P < 0.01 vs. Normal. For **F**, *P < 0.05 and **P < 0.01 vs. pre-infection. For **H**, *P < 0.05 and **P < 0.01 vs. the corresponding WT LV-Ctrl group. For **I**, **P < 0.01 vs. LV-Ctrl. Correlation analyses in **B** and **E** were performed using linear regression, and R² values are shown in the plots.

To identify potential downstream targets of miR-10b-3p, TargetScan analysis was combined with differential expression profiling from the GEO dataset GSE53819. Among the overlapping genes, three candidate targets were identified: *DST*, *PRAME*, and *ITGAV* (Fig 3A). Correlation analysis showed that, among these three candidates, only *ITGAV* mRNA expression exhibited a strong and significant inverse association with miR-10b-3p levels in NPC clinical specimens (Fig 3B), suggesting that *ITGAV* was the most likely downstream candidate for further validation. qPCR analysis further confirmed that *ITGAV* mRNA was markedly elevated in EBV-positive NPC tissues compared with non-malignant nasopharyngeal samples (Fig 3C), consistent with the bioinformatic prediction. At the protein level, IHC staining showed variable ITGAV expression among NPC tumor tissues (Fig 3D). Semi-quantitative scoring revealed a significant inverse correlation between miR-10b-3p expression and ITGAV IHC scores (Fig 3E). These data indicate that ITGAV expression is elevated in EBV-associated NPC tissues and inversely associated with miR-10b-3p abundance. To further verify whether EBV infection contributes to this regulatory pattern, EBV-negative cell lines NP69, SUNE-1, CNE-1, and HK1 were infected with the EBV M81 strain. *ITGAV* mRNA levels were significantly increased upon EBV infection in all four cell lines, whereas the endogenously EBV-positive C666-1 cells showed no notable change (Fig 3F). In parallel with the observed reduction in miR-10b-3p after EBV infection, these findings suggest that EBV-associated miR-10b-3p suppression may contribute to *ITGAV* upregulation in NPC cells. Together, these results support an inverse association between miR-10b-3p and *ITGAV* expression in EBV-positive NPC and provide the basis for subsequent mechanistic validation of their direct interaction.

### miR-10b-3p directly targets *ITGAV* and suppresses its expression

The predicted miR-10b-3p binding sites within the 3′-UTR of *ITGAV* were identified using the TargetScan algorithm and experimentally evaluated by dual-luciferase reporter assays (Fig 3G). Co-transfection of the WT *ITGAV* 3′-UTR reporter with LV-miR-10b significantly reduced luciferase activity in C666-1 and SUNE-1 cells compared with LV-Ctrl (Fig 3H). In C666-1 and SUNE-1 cells, miR-10b-3p overexpression significantly reduced luciferase activity of the WT *ITGAV* 3′-UTR reporter, whereas inhibition of endogenous miR-10b-3p in CNE-1 cells significantly increased reporter activity (Fig 3H). Neither treatment affected luciferase activity derived from the MUT *ITGAV* 3′-UTR reporter, confirming a sequence-specific interaction between miR-10b-3p and the *ITGAV* 3′-UTR. To determine whether miR-10b-3p regulates endogenous *ITGAV* expression, qPCR and Western blot analyses were performed following miR-10b-3p overexpression or inhibition. Ectopic expression of miR-10b-3p markedly reduced *ITGAV* mRNA and protein levels in C666-1 cells and EBV-infected SUNE-1 cells, whereas miR-10b-3p suppression markedly enhanced *ITGAV* mRNA and protein levels in the EBV-negative NPC cell lines CNE-1 and HK1 (Fig 3I, 3J). Collectively, these data provide direct experimental evidence that *ITGAV* is a direct target of miR-10b-3p, and that miR-10b-3p post-transcriptionally suppresses *ITGAV* expression through direct binding to its 3′-UTR. These findings provide a mechanistic link between EBV-associated miR-10b-3p downregulation and *ITGAV* upregulation in NPC cells.

### miR-10b-3p suppresses STAT5 and ERK1/2 signaling via ITGAV inhibition

Given the established role of ITGAV in activating proliferative signaling, downstream pathway modulation was examined. Overexpression of miR-10b-3p in C666-1 and EBV-infected SUNE-1 cells led to decreased phosphorylation of STAT5 and ERK1/2 (Fig 3K). Importantly, forced expression of ITGAV in miR-10b-3p-overexpressing cells partially restored STAT5 and ERK1/2 activation, supporting the conclusion that miR-10b-3p regulates these pathways through ITGAV. Collectively, these findings indicate that miR-10b-3p negatively regulates STAT5 and ERK1/2 signaling in an ITGAV-dependent manner. This pathway-level suppression provides a mechanistic basis for the inhibitory effects of miR-10b-3p on NPC cell proliferation and metastatic phenotypes.

### *ITGAV* partially mediates the tumor-suppressive effects of miR-10b-3p

**Fig 4:**
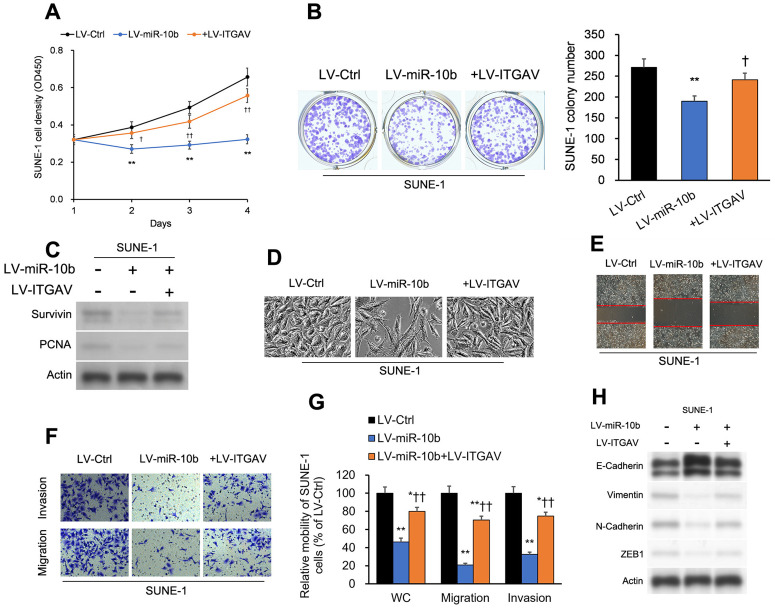
ITGAV re-expression partially rescues miR-10b-3p-mediated suppression of proliferation, migration, invasion, and EMT-associated changes in NPC cells. EBV-infected SUNE-1 cells were transfected with LV-Ctrl, LV-miR-10b, or LV-miR-10b + LV-ITGAV. LV-miR-10b denotes the miR-10b-3p overexpression construct, whereas LV-miR-10b + LV-ITGAV denotes a single dual-expression lentiviral construct expressing both miR-10b-3p and ITGAV. **(A)** Cell proliferation was determined using the CCK-8 assay. **(B)** Representative colony formation images and quantification of colony numbers. **(C)** Representative immunoblots showing the protein levels of the proliferation-associated markers Survivin and PCNA. **(D)** Representative phase-contrast images showing cell morphological changes. **(E)** Representative wound healing images used to assess cell migration. **(F)** Representative images from Transwell migration and Matrigel invasion assays. **(G)** Quantification of wound closure, migration, and invasion, normalized to the LV-Ctrl group. **(H)** Representative immunoblots showing the expression of EMT-associated markers E-cadherin, vimentin, N-cadherin, and ZEB1. For **A, B**, and **G**, data are shown as mean ± SEM from three independent biological experiments, each with three technical replicates. Immunoblots in **C** and **H** are representative of three independent experiments. *P < 0.05 and **P < 0.01 vs. LV-Ctrl; †P < 0.05 and ††P < 0.01 vs. LV-miR-10b. Statistical significance was analyzed using one-way ANOVA, except for the time-course proliferation assay in **A**, which was analyzed as described in the Methods.

To determine whether the biological effects of miR-10b-3p are functionally mediated, at least in part, through ITGAV, EBV-infected SUNE-1 cells were transfected with LV-Ctrl, LV-miR-10b, or LV-miR-10b + LV-ITGAV, a single dual-expression lentiviral construct expressing both miR-10b-3p and ITGAV (S3 Fig). Overexpression of ITGAV partially rescued the suppressive effects of miR-10b-3p on cell proliferation and colony-forming capacity, accompanied by partial recovery of proliferation-associated marker expression (Fig 4A–4D). Cell viability and clonogenic indices in the rescue group were partially restored toward control levels, indicating that the inhibitory activity of miR-10b-3p is mediated, at least in part, through ITGAV repression. Functional assays further showed that ITGAV overexpression partially rescued miR-10b-3p-induced suppression of migration and invasion, as reflected by increased wound closure and higher numbers of migrated and invaded cells in Transwell assays (Fig 4E–4G). At the molecular level, Western blot analysis showed that ITGAV overexpression partially reversed miR-10b-3p-mediated upregulation of E-cadherin and partially restored the expression of the mesenchymal markers N-cadherin, ZEB1, and Vimentin (Fig 4H). Collectively, these results support ITGAV as an important downstream effector that mediates the tumor-suppressive actions of miR-10b-3p in this EBV-positive NPC model. The partial functional restoration of proliferation, motility, and EMT phenotypes by ITGAV reintroduction indicates that miR-10b-3p exerts its antitumor effects, at least in part, by targeting the ITGAV-dependent STAT5 and ERK1/2 signaling axis.

## Discussion

EBV is among the most prevalent human viruses and has been implicated in the oncogenesis of several malignancies, including NPC, Burkitt’s lymphoma, Hodgkin’s lymphoma, nasal NK/T-cell lymphoma, and gastric carcinoma [22–24]. Dysregulation of host miRNAs is an important feature of EBV-associated cancers and represents one mechanism through which EBV can reshape host-cell regulatory networks during malignant transformation [19,25,26]. In this study, miR-10b-3p expression was significantly reduced in EBV-positive NPC tumors compared with non-malignant nasopharyngeal tissues. Functional analyses showed that miR-10b-3p suppresses NPC cell proliferation, EMT-associated changes, migration, and invasion *in vitro*, and reduces tumor growth and lung metastatic burden *in vivo*. These effects are mediated, at least in part, by direct repression of ITGAV, followed by attenuation of STAT5 and ERK1/2 signaling. Collectively, these findings suggest that EBV-associated suppression of miR-10b-3p may contribute to NPC progression by relieving miR-10b-3p-mediated restraint on ITGAV-dependent signaling.

The present work is distinctive in focusing on the 3p arm of the miR-10b locus, a strand whose function in EBV-associated NPC has remained poorly defined. Previous studies in NPC have mainly focused on miR-10b-5p, which can be induced by EBV-encoded LMP1 and has been implicated in metastasis through a Twist1-associated program [18]. The two mature strands originate from the same pre-miRNA hairpin but have distinct seed sequences and may regulate largely distinct target repertoires. In contrast to the previously described oncogenic role of miR-10b-5p, the present data indicate that miR-10b-3p is suppressed in the context of EBV infection and exerts tumor-suppressive effects. This strand-specific difference suggests that the biological output of the miR-10b locus in EBV-associated NPC may reflect a balance between 5p-associated pro-metastatic activity and 3p-mediated tumor restraint. EBV may shift this balance toward malignancy by favoring the previously reported oncogenic 5p program while suppressing the tumor-suppressive 3p strand. To our knowledge, this study provides the first mechanistic characterization of miR-10b-3p in EBV-associated NPC and identifies *ITGAV* as a direct functional target of this strand.

miRNAs typically regulate gene expression by binding to complementary sequences in the 3′-UTR of target mRNAs, thereby modulating translation or mRNA stability [27,28]. Similar miRNA-centered regulatory axes have also been implicated in cancer progression; for example, repression of miR-218-5p was reported to release the expression of the oncogenic effector USP32 and thereby promote hepatocellular carcinoma development [29]. In this study, bioinformatic analyses nominated *ITGAV* as a candidate target, and dual-luciferase reporter assays validated *ITGAV* as a direct target of miR-10b-3p. *ITGAV* encodes the integrin alpha V subunit, which heterodimerizes with multiple β subunits, including β1, β3, and β5, to form αv-containing integrins that regulate cell adhesion, migration, and intracellular signaling [30,31]. Overexpression of ITGAV has been reported in several human cancers and is frequently associated with aggressive phenotypes and poor clinical outcomes [32–36]. *ITGAV* upregulation has also been linked to enhanced cell motility and invasiveness through pathways involving focal adhesion kinase, NF-κB, and MAPK signaling [37]. The present findings show elevated ITGAV expression in EBV-positive NPC tissues and EBV-infected cell models, together with an inverse association between ITGAV and miR-10b-3p expression. In the current study, miR-10b-3p overexpression decreased phosphorylation of STAT5 and ERK1/2, whereas ITGAV re-expression partially restored activation of these pathways. These observations support a model in which EBV-associated miR-10b-3p downregulation promotes NPC progression, at least in part, through ITGAV-dependent activation of STAT5 and ERK1/2 signaling.

EBV-associated activation of NF-κB signaling has been linked to broad alterations in host transcriptional and post-transcriptional networks [38]. Enhanced NF-κB activity is known to upregulate *ITGAV* expression in several cancers, suggesting that NF-κB-dependent transcriptional regulation may also contribute to *ITGAV* induction in NPC [37]. In this context, EBV-associated suppression of miR-10b-3p may contribute to *ITGAV* upregulation, whereas additional miR-10b-3p-independent mechanisms, such as NF-κB-dependent transcriptional regulation, remain possible and require direct validation. Such convergence between viral and cellular pathways could reinforce oncogenic signaling, although this possibility requires direct experimental validation. Future studies should clarify which EBV latent factors are responsible for miR-10b-3p repression and determine whether NF-κB-dependent transcriptional regulation cooperates with loss of miR-10b-3p-mediated post-transcriptional control to increase *ITGAV* expression.

A further implication of the present findings relates to EBV latency in NPC. miR-10b-3p overexpression reduced ZEB1, a mesenchymal marker and transcriptional regulator involved in EMT. Beyond its role in EMT, ZEB1 has been reported to repress the EBV immediate-early gene *BZLF1* by binding to a silencing element within the *BZLF1* promoter (Zp) [39,40]. Therefore, EBV-associated suppression of miR-10b-3p could potentially increase ZEB1 expression and reinforce repression of *BZLF1*, thereby favoring maintenance of latency. This possibility provides a plausible link between miR-10b-3p dysregulation, EMT-associated remodeling, and EBV latency control in NPC. However, this proposed miR-10b-3p/ZEB1/*BZLF1* connection remains speculative and should be tested directly by assessing ZEB1 occupancy at Zp, *BZLF1* expression, and spontaneous or induced lytic reactivation after miR-10b-3p restoration.

Several limitations should be noted. First, although EBV infection was associated with reduced miR-10b-3p expression, the specific EBV latent factor and molecular mechanism responsible for miR-10b-3p repression were not defined. Second, ITGAV re-expression produced only partial rescue of miR-10b-3p-mediated phenotypes, indicating that additional miR-10b-3p targets may contribute to its tumor-suppressive activity. Third, the tail-vein model used in this study primarily reflects experimental lung metastatic colonization and does not fully recapitulate the complete process of spontaneous metastasis from a primary NPC lesion. Finally, the clinical cohort was limited in size, and the prognostic value of the miR-10b-3p/ITGAV axis requires validation in larger patient cohorts with survival and treatment-response data.

In summary, this study defines an EBV-regulated miR-10b-3p/ITGAV/STAT5-ERK1/2 signaling axis that links EBV-associated host miRNA dysregulation to proliferative and metastatic phenotypes in NPC (S4 Fig). The data indicate that loss of miR-10b-3p relieves repression of ITGAV, thereby promoting STAT5 and ERK1/2 activation and contributing to tumor growth and lung metastatic colonization. These findings provide functional evidence for the previously under-characterized 3p arm of the miR-10b locus and suggest that restoration of miR-10b-3p activity or inhibition of ITGAV-dependent signaling may represent potential therapeutic directions for EBV-associated NPC. Future work should define the viral mechanisms responsible for miR-10b-3p suppression, test the proposed ZEB1/*BZLF1* latency-related mechanism, use single-cell and spatial omics to clarify the cellular context of the miR-10b-3p/ITGAV axis, and evaluate axis-directed interventions in additional preclinical models [41].

## Materials and methods

### Ethics statement

The retrospective use of archived human specimens in this study was approved by the Ethics Committee of the First People’s Hospital of Yunnan Province (KHLL2025-KY172). Written informed consent had been obtained from all participants at the time of sample collection. All animal procedures complied with the guidelines set forth in the NIH Guide for the Care and Use of Laboratory Animals and had been approved by the Animal Ethics Committee of the Kunming Medical University (kmmu20221570).

### Tissue specimens

Undifferentiated NPC tumor specimens were collected from adult EBV-positive patients, and non-malignant nasopharyngeal tissues were obtained from demographically comparable individuals with chronic nasopharyngeal inflammation who served as controls. All specimens were obtained at the First People’s Hospital of Yunnan Province between January 2017 and December 2018. Inclusion criteria were as follows: (i) no prior chemotherapy or radiotherapy before tissue collection; (ii) written informed consent obtained before sample collection; (iii) for NPC cases, EBV positivity confirmed by BamHI-W qPCR-based plasma cell-free EBV DNA assay (cf EBV DNA ≥ 100 copies/mL) and serum VCA/IgA antibody geometric mean titer (GMT ≥ 1:100); (iv) for controls, no detectable plasma cell-free EBV DNA and negative VCA/IgA serology; and (v) histopathological diagnosis of undifferentiated NPC or non-malignant inflammatory nasopharyngeal tissue confirmed by two independent pathologists. A total of 30 NPC cases (median age, 47 years; range, 20–79; 63% male) and 30 demographically comparable controls (median age, 46 years; range, 19–80; 67% male) meeting the above criteria were included in this study. All tissues were formalin-fixed and paraffin-embedded (FFPE) for subsequent histological and molecular analyses. Written informed consent was obtained from all participants before sample collection.

### Cell lines and culture

Human embryonic kidney 293T (HEK293T), SV40-immortalized human nasopharyngeal epithelial NP69 [42], EBV-negative NPC cell lines CNE-1, SUNE-1, and HK1 [43], and the EBV-positive NPC cell line C666-1 [20] were obtained from the Cell Bank of the Chinese Academy of Sciences (Shanghai, China). Cells were maintained in RPMI-1640 medium (Gibco, USA) supplemented with 10% fetal bovine serum (FBS; Gibco, USA), 100 U/mL penicillin, and 100 µg/mL streptomycin at 37 °C in a humidified incubator containing 5% CO₂. All cell lines were routinely tested and confirmed to be free of mycoplasma contamination before use.

### Production, infection, and enrichment of EBV-infected cells

Recombinant EBV particles were produced in HEK293T cells harboring the EBV M81 bacterial artificial chromosome (BACMID), which carries an enhanced green fluorescent protein (eGFP) reporter cassette and a hygromycin resistance gene, as previously described [44,45]. Briefly, HEK293T cells were transfected with 1 µg of BACMID DNA per 4 × 10⁵ cells using Metafectene reagent (Biontex, Germany), and EBV BACMID-containing clones were selected under hygromycin B (100 µg/mL) with confirmation of GFP fluorescence. To induce lytic replication, stable clones were co-transfected with *BZLF1* (p509) and *BALF4* (pRA) expression plasmids using Metafectene reagent. Culture supernatants were collected 72 h after induction, clarified by low-speed centrifugation, filtered through 0.45-µm membranes, and concentrated by centrifugation at 20,000 × g for 2 h at 4 °C. Viral titers were determined by quantitative PCR (qPCR) targeting the *BALF5* gene and expressed as viral genome equivalents per mL [44].

For infection of nasopharyngeal epithelial and NPC cell lines, EBV-negative NP69, SUNE-1, CNE-1, and HK1 cells were seeded at 1 × 10⁶ cells per well in 6-well plates and exposed to concentrated virus at a dose of 200 viral genome equivalents per cell in the presence of 8 µg/mL polybrene (Sigma-Aldrich, USA) for 72 h [45]. Because direct EBV infection of epithelial cells is inefficient, infected cells were enriched by a two-step procedure. First, GFP-positive cells were isolated by fluorescence-activated cell sorting on a BD FACSAria II cell sorter (BD Biosciences, USA) using 488 nm excitation and a 530/30 nm emission filter, with EBV-negative parental cells serving as the GFP-negative gating control. Sorted GFP-positive populations were then maintained under hygromycin B selection (100 µg/mL; Invitrogen, USA) for 5 days to further enrich cells carrying the EBV BACMID episome. Enriched cells were expanded for an additional 3–7 days in selection-free medium before downstream assays. Infection efficiency and EBV retention were assessed by GFP fluorescence microscopy, and EBV positivity was verified by qPCR for the viral *BALF5* gene and by RT-qPCR for the latent transcripts *EBER1/2* and *LMP1*. Enriched EBV-infected cells were used within 2 weeks of sorting to minimize episome loss, and parallel uninfected parental cultures were maintained as matched controls. For downstream functional assays, miR-10b-3p overexpression experiments were performed in EBV-positive C666-1 cells and EBV-infected SUNE-1 cells. These two cell models were characterized by low endogenous miR-10b-3p expression. By contrast, inhibition of endogenous miR-10b-3p was performed in EBV-negative CNE-1 and HK1 cells, which showed relatively high endogenous miR-10b-3p expression. All EBV work was performed under BSL-2 containment in accordance with institutional biosafety guidelines.

### Expression vector construction and transfection

An engineered stem-loop hsa-miR-10b-3p precursor sequence was cloned into the pLVTHM lentiviral vector (Addgene plasmid #12247) to generate LV-miR-10b. For rescue experiments, the full-length human *ITGAV* cDNA sequence (NM_002210) was inserted into the LV-miR-10b backbone to generate LV-miR-10b + LV-ITGAV, a single dual-expression lentiviral construct expressing both miR-10b-3p and ITGAV. To inhibit miR-10b-3p expression, an antisense stem-loop precursor sequence of hsa-miR-10b-3p was cloned into the same vector backbone and designated LV-anti-miR-10b. In these constructs, the miRNA precursor sequence was placed under the control of the polymerase III H1 promoter, whereas the *ITGAV* cDNA was driven by the eukaryotic elongation factor 1-alpha (eEF1a1)/central polypurine tract (cPPT) promoter element. An empty pLVTHM vector served as the negative control (LV-Ctrl). For *in vivo* lung metastasis assays, the firefly luciferase (Luc) cassette from the pGL3 vector (Promega, USA) was subcloned into the LV-Ctrl and LV-miR-10b constructs to generate LV-Ctrl-Luc and LV-miR-10b-Luc, respectively. The Luc cassette was also regulated by the eEF1a1/cPPT promoter. All recombinant constructs were confirmed by restriction digestion and DNA sequencing. Expression vectors were transfected into recipient NPC cells using Lipofectamine 3000 Transfection Reagent (Invitrogen, USA) according to the manufacturer’s protocol. Transfection efficiency was confirmed by qPCR and fluorescence microscopy.

### Real-time quantitative PCR (qPCR)

Total RNA was extracted from NPC tissues and cell lines using TRIpure reagent (Roche, Switzerland) according to the manufacturer’s instructions. The HiFiScript cDNA Synthesis Kit (CoWin Biotech, China) and Bulge-Loop microRNA-specific reverse transcription primers (Bioray, China) were used to reverse transcribe RNA into cDNA. For qPCR analysis, Exiqon miRCURY PCR primers specific for hsa-miR-10b-3p (cat. no. 204514) and U6 snRNA (cat. no. 203907) were used. Primers for firefly luciferase (Luc) were used as described previously [44]. Primers for mRNA quantification were obtained from Origene (Beijing, China) and are listed in S1 Table. qPCR reactions were carried out using ExiLENT SYBR Green Master Mix (Exiqon, Denmark) on a CFX384 Real-Time PCR Detection System (Bio-Rad, USA). U6 snRNA and GAPDH served as internal normalization controls for miRNA and mRNA quantification, respectively. Relative expression levels were determined using the 2^⁻ΔΔCt^ method.

### Western blot analysis

Total protein was extracted from cultured cells using protein extraction reagents from Millipore (USA) in the presence of protease and phosphatase inhibitors, according to the manufacturer’s protocol. Protein concentrations were determined using a BCA protein assay. Equal amounts of protein were resolved by SDS-PAGE and transferred to PVDF membranes. Membranes were blocked with 5% skim milk in TBST and incubated overnight at 4 °C with the following primary antibodies: anti-PCNA (#13119, Cell Signaling Technology [CST]), anti-Survivin (#2808T, CST), anti-E-cadherin (ab15148, Abcam), anti-N-cadherin (#13116T, CST), anti-Vimentin (#5741T, CST), anti-ZEB1 (#3396T, CST), anti-phospho-STAT5^Tyr694^ (#9359, CST), anti-STAT5 (#94205, CST), anti-phospho-ERK1/2^Thr202/Tyr204^ (#4377, CST), anti-ERK1/2 (#4695, CST), anti-ITGAV (ab179475, Abcam), and anti-β-actin (#4967, CST; loading control). After washing, membranes were incubated with horseradish peroxidase-conjugated secondary antibodies, and signals were visualized using enhanced chemiluminescence (Thermo Fisher Scientific, USA).

### Candidate target gene prediction and expression filtering

Candidate targets of hsa-miR-10b-3p were predicted using the TargetScanHuman database (http://www.targetscan.org/vert_80/) with a cumulative weighted context++ score threshold of ≤ -0.50. To identify genes upregulated in NPC, microarray data from the GEO dataset GSE53819 were analyzed. This dataset includes expression profiles from 18 chemotherapy-naive NPC tissues and 18 non-cancerous nasopharyngeal tissues [46]. Genes with log_2_ fold-change ≥ 1.0 and adjusted P < 0.05 were considered significantly upregulated. Overlapping genes between the predicted target list and the NPC-upregulated gene list were then identified for subsequent validation.

### Dual-luciferase reporter assay

The full-length wild-type (WT) 3′ untranslated region (UTR) sequence of human *ITGAV* was obtained from the University of California, Santa Cruz (UCSC) Genome Browser (hg18 assembly). Two putative hsa-miR-10b-3p binding sites predicted by the TargetScan database were mutated using the QuikChange Lightning Site-Directed Mutagenesis Kit (Agilent Technologies, USA) to generate the mutant (MUT) *ITGAV* 3′-UTR reporter. Both WT and MUT *ITGAV* 3′-UTR fragments were amplified and subcloned into the pGL4 luciferase reporter vector (Promega, USA). After sequence verification, 2 µg of each reporter plasmid was co-transfected into NPC cells with LV-Ctrl, LV-miR-10b, or LV-anti-miR-10b expression constructs, as indicated. A Renilla luciferase plasmid was co-transfected in each well as an internal normalization control. Twenty-four hours after transfection, cells were lysed, and luciferase activity was measured using the Dual-Luciferase Reporter Assay System (Promega, USA) according to the manufacturer’s instructions. Firefly luciferase activity was normalized to Renilla luciferase activity to calculate relative luciferase activity.

### Cell proliferation and colony formation assays

Cell proliferation was evaluated using the Cell Counting Kit-8 (CCK-8; Dojindo, Japan). Briefly, EBV-infected SUNE-1, EBV-negative CNE-1, and EBV-positive C666-1 cells were seeded into 96-well plates. EBV-infected SUNE-1 and CNE-1 cells were seeded at 5 × 10³ cells per well, whereas C666-1 cells were seeded at 1 × 10⁴ cells per well, in 100 µL of culture medium. Cells were incubated for 24, 48, 72, and 96 h. At each time point, 10 µL of CCK-8 reagent was added to each well and incubated for an additional 2 h at 37 °C. Absorbance was measured at 450 nm using a microplate reader (Bio-Rad, USA), and cell viability was expressed as absorbance at 450 nm or normalized to the corresponding control group as indicated. For colony formation assays, the indicated NPC cells were seeded into six-well plates at 800 cells per well and cultured for 14 days, with the medium replaced every three days. At the end of incubation, cells were fixed with methanol for 20 min and stained with 0.5% crystal violet. Colonies containing ≥50 cells were counted under a microscope.

### Wound healing and Transwell migration/invasion assays

Cell migration was evaluated using a wound healing assay as previously described [21]. Briefly, NPC cells were seeded into six-well plates and grown to 95–100% confluence. The culture medium was replaced with serum-free medium 24 h before scratching to minimize the contribution of cell proliferation to wound closure. A uniform linear wound was created across the cell monolayer using a sterile pipette tip, and detached cells were removed by washing twice with PBS. Cells were then incubated in serum-free medium for an additional 48 h, and wound closure was photographed at 0 and 48 h using an inverted microscope. Wound closure was quantified based on the reduction in wound width between 0 and 48 h.

Cell migration and invasion were further assessed using Transwell assays (Corning, USA). For migration assays, uncoated Transwell inserts with 8-µm pores were used, whereas for invasion assays, inserts were pre-coated with 20 µg/well Matrigel (BD Biosciences, USA). EBV-infected SUNE-1 and EBV-negative CNE-1 cells were seeded at 5 × 10⁴ cells per insert, whereas EBV-positive C666-1 cells were seeded at 1 × 10⁵ cells per insert. Cells were suspended in 250 µL of serum-free medium and added to the upper chambers, and 500 µL of medium containing 20% FBS was added to the lower chambers as a chemoattractant. Cells were incubated for 24 h for SUNE-1 cells and 48 h for CNE-1 and C666-1 cells at 37 °C. Non-migrated or non-invaded cells on the upper surface of the membrane were gently removed. Cells on the lower surface of the membrane were fixed with 10% formalin, stained with 0.5% crystal violet for 20 min, and counted under an inverted microscope in five randomly selected fields per membrane.

### Xenograft tumor growth and experimental lung metastasis models

Female BALB/c nude mice, 4–7 weeks old and weighing 20–25 g, were purchased from Shanghai SLAC Laboratory Animal Co., Ltd. (Shanghai, China). For the subcutaneous xenograft model, EBV-infected SUNE-1 cells transfected with LV-miR-10b or the corresponding control vector (LV-Ctrl) were expanded in culture, and 1 × 10⁶ cells were injected subcutaneously into the dorsal flank of each mouse (n = 9 mice per group). Tumor size was measured every three days using calipers, and tumor volume was calculated as length × width²/2. At 15 days after inoculation, mice were euthanized, and xenograft tumors were excised, weighed, and processed for hematoxylin and eosin (H&E) staining.

For the experimental lung metastasis model, 5 × 10⁵ EBV-infected SUNE-1 cells transfected with LV-miR-10b-Luc or the control vector LV-Ctrl-Luc were injected via the tail vein (n = 9 mice per group). At five weeks after injection, mice received an intraperitoneal injection of VivoGlo Luciferin (150 mg/kg; Promega, USA), followed by bioluminescence imaging using an *in vivo* imaging system (Caliper Life Sciences, USA) to quantify lung metastatic burden. Mice were subsequently euthanized, and lungs were collected, fixed in 10% formalin, embedded in paraffin, sectioned, and stained with H&E. Macroscopic lung metastases were quantified as a percentage of the total lung surface area, and microscopic metastatic foci were calculated as a percentage of the total lung sectional area.

### Immunohistochemistry (IHC)

ITGAV protein expression in FFPE tissue sections was assessed by IHC staining. Briefly, 4-µm FFPE tissue sections were deparaffinized, rehydrated, subjected to antigen retrieval, and incubated with anti-ITGAV antibody (Abcam, ab154020) for 1 h at 37 °C. After washing, sections were incubated with HRP-conjugated secondary antibody (Elivision Super HRP Kit, Maixin-Bio, China) for 30 min, followed by diaminobenzidine chromogenic detection and hematoxylin counterstaining. Staining intensity and the proportion of positive cells were semi-quantitatively scored as previously described [47]. Staining intensity was scored as 0 = negative, 1 = weak, 2 = moderate, and 3 = strong. The percentage of positive cells was scored as 0 = ≤25%, 1 = 25.1-50%, 2 = 50.1-75%, and 3 = > 75%. The final IHC score was calculated by multiplying the staining intensity score by the percentage score, yielding a total score ranging from 0 to 9. Scores of 0–3, 4–6, and 7–9 were classified as low, medium, and high ITGAV expression, respectively.

### Statistical analysis

All quantitative data are presented as mean ± standard error of the mean (SEM). Statistical analyses were performed using SPSS version 21.0 (IBM Corp.) and GraphPad Prism 8.0 (GraphPad Software). Differences between two groups were evaluated using an unpaired, two-tailed Student’s t-test, whereas multiple group comparisons were conducted using one-way ANOVA followed by Tukey’s post hoc test. Correlations between continuous variables were assessed using Pearson correlation analysis or linear regression analysis, as appropriate. For correlation plots with fitted regression lines, the coefficient of determination (R^2^) and corresponding P values are shown. A P-value < 0.05 was considered statistically significant.

## Supporting information

S1 Fig Validation of miR-10b-3p modulation in transfected NPC cell lines. Expression of miR-10b-3p in the NPC cell lines (A) C666-1, (B) SUNE-1, (C) CNE-1, and (D) HK1 following transfection with LV-Ctrl, LV-miR-10b vectors, or LV-anti-miR-10b as indicated. The housekeeping control used was U6 snRNA. n = 3 biological replicates × 3 technical replicates. Data are represented as mean ± SEM. *P < 0.05, **P < 0.01 vs. LV-Ctrl [unpaired Student’s t-test].
(DOCX)

S2 Fig Validation of miR-10b-3p and Luc overexpression in transfected SUNE-1 cells. Expression of (A) miR-10b-3p and (B) Luc mRNA in non-transfected (NT) SUNE-1 cells and following transfection with LV-Ctrl-Luc or LV-miR-10b-Luc vectors. U6 snRNA was used for miR-10b-3p normalization, and GAPDH was used for Luc mRNA normalization. n = 3 biological replicates × 3 technical replicates. Data are represented as mean ± SEM. *P < 0.05, **P < 0.01 vs. NT [unpaired Student’s t-test].
(DOCX)

S3 Fig Validation of miR-10b-3p and *ITGAV* overexpression in transfected C666-1 and SUNE-1 cells. miR-10b-3p and *ITGAV* mRNA expression in (A, B) C666-1 cells and (C, D) SUNE-1 cells following transfection with LV-Ctrl, LV-miR-10b, or the LV-miR-10b + LV-ITGAV dual-expression construct. U6 snRNA was used for miR-10b-3p normalization, and GAPDH was used for ITGAV mRNA normalization. n = 3 biological replicates × 3 technical replicates. Data are represented as mean ± SEM. *P < 0.05, **P < 0.01 vs. LV-Ctrl, †P < 0.05, ††P < 0.01 vs. LV-miR-10b [one-way ANOVA].
(DOCX)

S4 Fig Schematic overview of the EBV-regulated miR-10b-3p/ITGAV axis in nasopharyngeal carcinoma. EBV infection is associated with reduced miR-10b-3p expression in nasopharyngeal epithelial and NPC cells. Decreased miR-10b-3p expression relieves repression of ITGAV, resulting in increased ITGAV expression and activation of downstream STAT5 and ERK1/2 signaling. This pathway contributes to enhanced cell proliferation, migration, invasion, epithelial-mesenchymal transition, tumor growth, and lung metastatic colonization in EBV-associated NPC.
(DOCX)

S1 Table The primers used in this study. (DOCX)
