# Supplementary figures and images for: Downregulation of miR-10b-3p by EBV promotes tumor growth and metastasis via ITGAV in nasopharyngeal carcinoma
Source: PLoS Pathog. 2026 Jun 9;22(6):e1014304. doi: 10.1371/journal.ppat.1014304 (PMC13249171; doi:10.1371/journal.ppat.1014304)

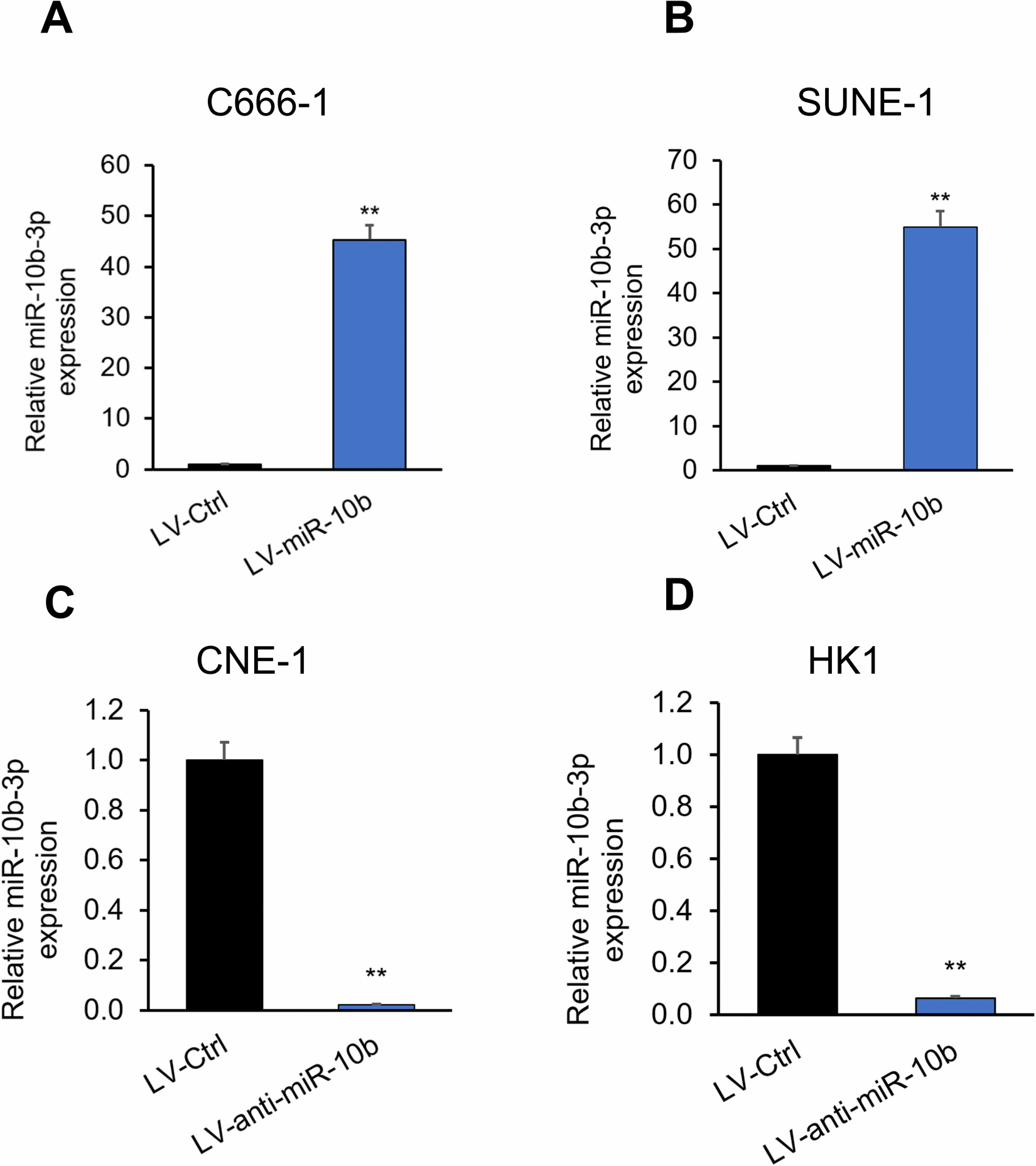

Supplement: S1 Fig — Expression of miR-10b-3p in the NPC cell lines (A) C666-1, (B) SUNE-1, (C) CNE-1, and (D) HK1 following transfection with LV-Ctrl, LV-miR-10b vectors, or LV-anti-miR-10b as indicated. The housekeeping control used was U6 snRNA. n = 3 biological replicates × 3 technical replicates. Data are represented as mean ± SEM. *P < 0.05, **P < 0.01 vs. LV-Ctrl [unpaired Student’s t-test]. (DOCX) [file ppat.1014304.s001.docx]

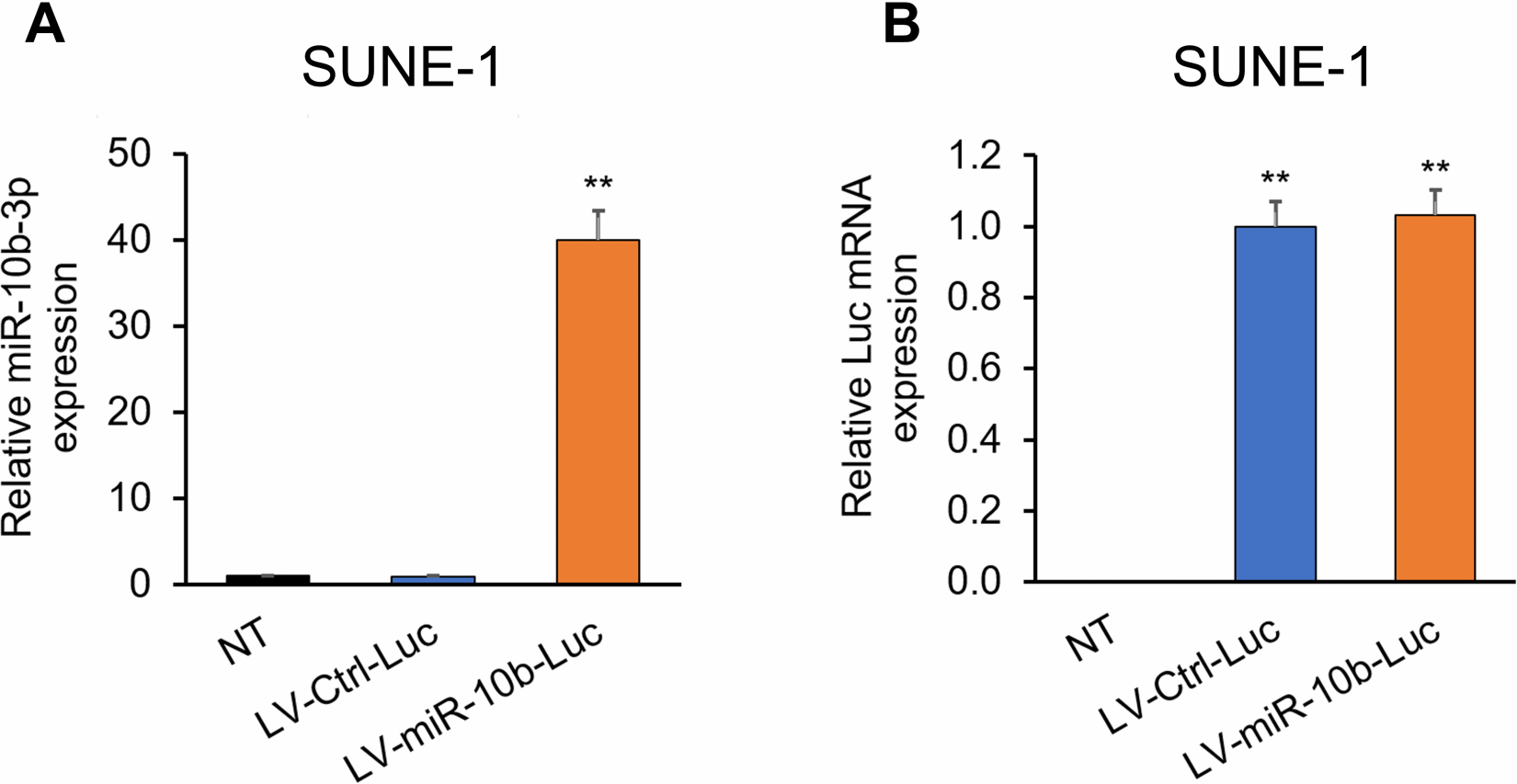

Supplement: S2 Fig — Expression of (A) miR-10b-3p and (B) Luc mRNA in non-transfected (NT) SUNE-1 cells and following transfection with LV-Ctrl-Luc or LV-miR-10b-Luc vectors. U6 snRNA was used for miR-10b-3p normalization, and GAPDH was used for Luc mRNA normalization. n = 3 biological replicates × 3 technical replicates. Data are represented as mean ± SEM. *P < 0.05, **P < 0.01 vs. NT [unpaired Student’s t-test]. (DOCX) [file ppat.1014304.s002.docx]

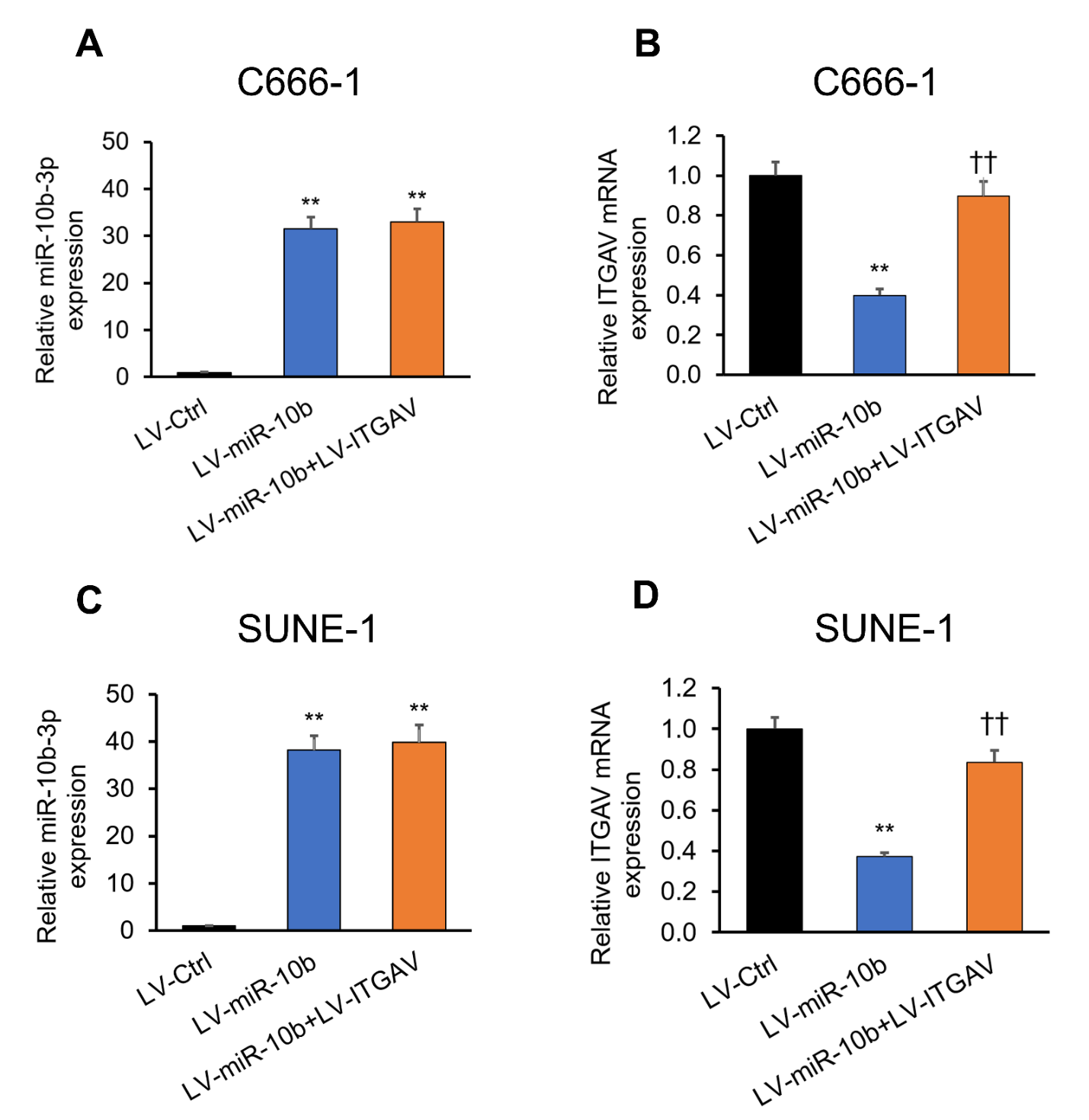

Supplement: S3 Fig — U6 snRNA was used for miR-10b-3p normalization, and GAPDH was used for ITGAV mRNA normalization. n = 3 biological replicates × 3 technical replicates. Data are represented as mean ± SEM. *P < 0.05, **P < 0.01 vs. LV-Ctrl, †P < 0.05, ††P < 0.01 vs. LV-miR-10b [one-way ANOVA]. (DOCX) [file ppat.1014304.s003.docx]

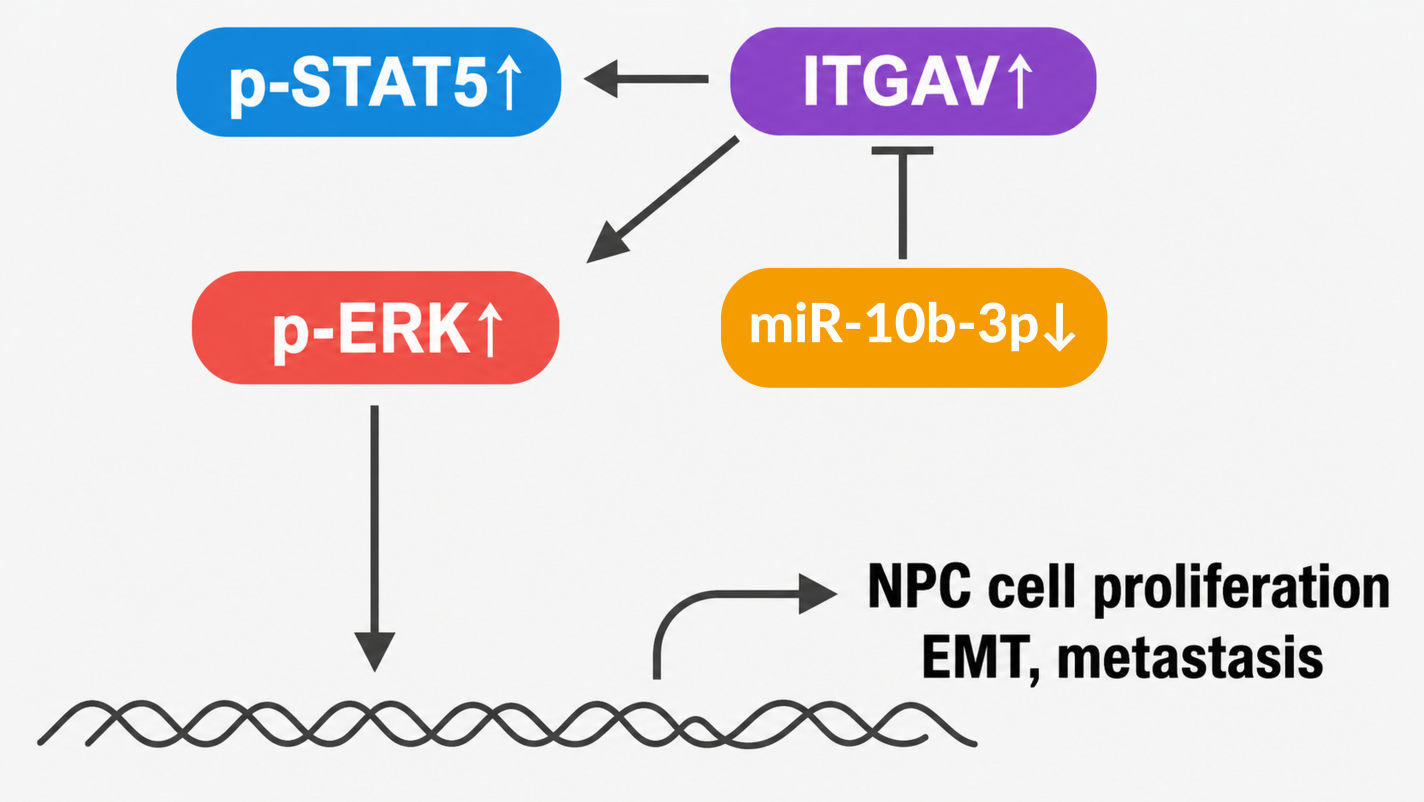

Supplement: S4 Fig — EBV infection is associated with reduced miR-10b-3p expression in nasopharyngeal epithelial and NPC cells. Decreased miR-10b-3p expression relieves repression of ITGAV, resulting in increased ITGAV expression and activation of downstream STAT5 and ERK1/2 signaling. This pathway contributes to enhanced cell proliferation, migration, invasion, epithelial-mesenchymal transition, tumor growth, and lung metastatic colonization in EBV-associated NPC. (DOCX) [file ppat.1014304.s004.docx]
