# Supplementary material for: Downregulation of miR-10b-3p by EBV promotes tumor growth and metastasis via ITGAV in nasopharyngeal carcinoma
Source: PLoS Pathog. 2026 Jun 9;22(6):e1014304. doi: 10.1371/journal.ppat.1014304 (PMC13249171; doi:10.1371/journal.ppat.1014304)
Supplement: S1 Table — (DOCX) [file ppat.1014304.s005.docx]

**S1 Table. The primers used in this study**

| **Primers** | **Sequence (5’-3’)** | **Size (bp)** |
| --- | --- | --- |
| *Luc*-F | CCGGGCGCGGTCGGTAAAG | 19 |
| *Luc*-R | CGGCGGGAAGTTCACCGGCG | 20 |
| *ITGAV*-F | AGGAGAAGGTGCCTACGAAGCT | 22 |
| *ITGAV*-R | GCACAGGAAAGTCTTGCTAAGGC | 23 |
| *DST*-F | CCAGGCACTCTGTGAGGATTTG | 22 |
| *DST*-R | CGACTGAAGGTGCTGGACATGA | 22 |
| *PRAME*-F | GGAGTGCTGATGAAGGGACAAC | 22 |
| *PRAME*-R | CAGTCCAGAAGTCCTGATGAGAG | 23 |
| *GAPDH*-F | GTCTCCTCTGACTTCAACAGCG | 22 |
| *GAPDH*-R | ACCACCCTGTTGCTGTAGCCAA | 22 |
